# Aesthetic and Functional Outcomes of the Innervated and Thinned Anterolateral Thigh Flap in Reconstruction of Upper Limb Defects

**DOI:** 10.1155/2014/489012

**Published:** 2014-11-16

**Authors:** Carlos Alberto Torres-Ortíz Zermeño, Javier López Mendoza

**Affiliations:** ^1^Plastic and Reconstructive Surgery, General Hospital Dr. Manuel Gea González, Calzada de Tlalpan No. 4800, 14080 Mexico City, DF, Mexico; ^2^Hand and Microsurgery Clinic, General Hospital Dr. Manuel Gea González, Calzada de Tlalpan No. 4800, 14080 Mexico City, DF, Mexico

## Abstract

*Background*. The anterolateral thigh (ALT) flap has been widely described in reconstruction of the upper extremity. However, some details require refinement to improve both functional and aesthetic results. *Methods*. After reconstruction of upper extremity defects using thinned and innervated ALT flaps, functional and aesthetic outcomes were evaluated with the QuickDASH scale and a Likert scale for aesthetic assessment of free flaps, respectively. *Results*. Seven patients with a mean follow-up of 11.57 months and average flap thickness of 5 mm underwent innervation by an end-to-end neurorrhaphy. The average percentage of disability (QuickDASH) was 21.88% with tenderness, pain, temperature, and two-point discrimination present in 100% of cases, and the aesthetic result gave an overall result of 15.40 (good) with the best scores in color and texture. *Conclusions*. Simultaneous thinning and innervation of the ALT flap lead to a good cosmetic result and functional outcome with a low percentage of disability, which could result in minor surgical procedures and better recovery of motor and sensory function. *Level of Evidence*. IV.

## 1. Introduction

The most appropriate procedure to correct a defect is currently chosen according to the best possible functional and aesthetic outcome. This represents a change in the reconstructive ladder, improving the functional and aesthetic results that can be achieved with currently free flaps.

The anterolateral thigh (ALT) flap was described in 1984 by Song et al. as a fasciocutaneous flap based on perforators of the descending branch of the lateral femoral circumflex artery. This artery runs caudally in the intermuscular septum between the rectus femoris and vastus lateralis, giving off multiple septocutaneous and musculocutaneous perforators. The flap dimensions vary according to whether it is based on a perforator vessel (up to 20 × 12 cm) or musculocutaneous vessels (up to 34 × 14 cm) [1–3].

Thinning and innervation of the ALT flap have been partially studied by several centers worldwide, but there are still discussions about the limit of thinning of the ALT flap and the prognosis when it is innervated [4], even when there are some studies about the vascular supply and the limits that its perforator vessel can irrigate [5].

In the field of the reconstruction of the upper extremity and, even more, in the hand, the ideal selected flap should meet certain requirements like tissue for replacing like area, thin and pliable flap for molding the hand contour, minimal donor-site morbidity, and sizable pedicle for microsurgical anastomosis; also, change of position intraoperatively should not be necessary [6].

Thinning of the ALT flap has become a popular choice in the reconstruction of soft tissues because it obtains a high-quality and less bulky tissue. The results of reconstruction are more functional and aesthetic and, in some cases, do not require additional debulking procedures [7].

Successful thinning of the ALT flap has been described in both Asian and Western patients. In 2003, Alkureishi et al. analyzed a series of 10 ALT flaps in Western patients using arterial markers and flap measures. Partial loss of subdermal plexus blood, enough to produce skin necrosis at the distal areas of the flap, was observed [8].

Reconstruction with the ALT flap is a commonly performed surgery, at least in our center. However, we believe that simultaneous thinning and innervation of the ALT flap improve the aesthetic result, promote early rehabilitation, improve the functionality of the limb, and recreate as much as possible the healthy tissue characteristics of the recipient zone. In addition, it preserves motor activity and the perceptions of touch, pain, and temperature in the reconstructed area.

Most published studies refer to the thinning or innervation of free flaps, but not the combination of these techniques. The purpose of this study was to evaluate the functional and aesthetic results of the ultrathin ALT flap and its innervation in soft tissue reconstruction in the upper extremity.

## 2. Material and Methods

We analyzed seven patients from June 2010 to May 2011 who were considered to be reconstructed by the herein-described method according to the criteria here exposed. Inclusion criteria were sequelae of previous trauma or acute trauma, by a sequential presentation of cases, whose defect was not a candidate for local or regional flap reconstruction, with exposure of deep structures, abundant scar tissue that generates the joint limitation to the flexion or extension, or the joint distortion. And our last inclusion criteria was the reconstruction of the limb with the recovery of the sensibility.

In this series, the indication for reconstruction was acute trauma in three patients, sequelae of crushing trauma in one patient, and burn sequelae in the remaining three patients (Table 1).

### 2.1. Surgical Technique

**Figure 1:**
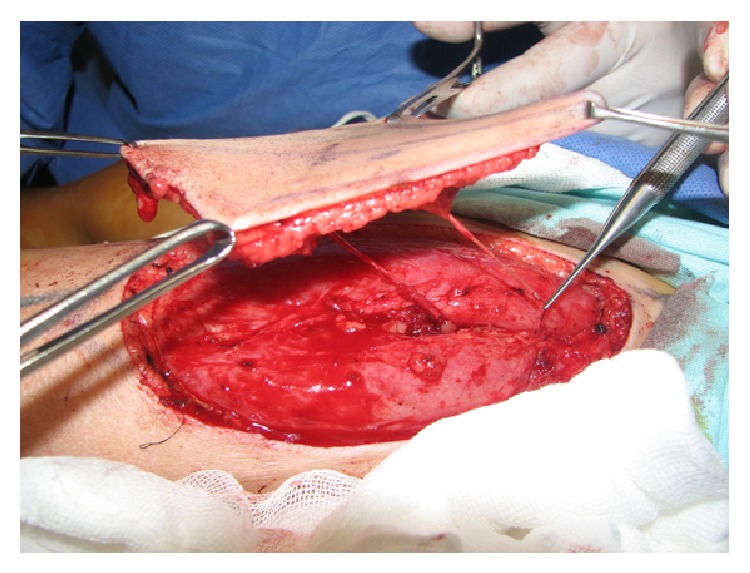
Thinned anterolateral thigh flap, with its vascular pedicle and the nerve.

The ALT flap was marked in the habitual manner, and the perforators vessels were identified preoperatively by Doppler US. The rise begins with blunt undermining from medial to lateral, until the localization of the major perforator vessel, preserving the superficial fascia, and a ratio around the perforator vessel of 2 cm to ensure flap survival. The dominant nerve (lateral femoral cutaneous nerve) was identified and preserved. The vascular pedicle was followed in a retrograde fashion to its origin to obtain a length of at least 7 to 10 cm for comfortable anastomoses. The thinning of the flap was performed with curved iris scissors, leaving a homogenous thickness in most of the flap, outside the security perforator ratio (Figure 1). The thickness of the flap was measured in all the flaps with a centimeters rule, 2 cm outside the security ratio. Vessel and nerve anastomoses were performed with 9-0 nylon, and the extremity was immobilized by a splint. All patients underwent thinning and innervation of the flap by neurorrhaphy to a sensory nerve adjacent to the reconstructed area, usually to one of the sensory branches of the radial nerve.

### 2.2. Evaluation

**Figure 2:**
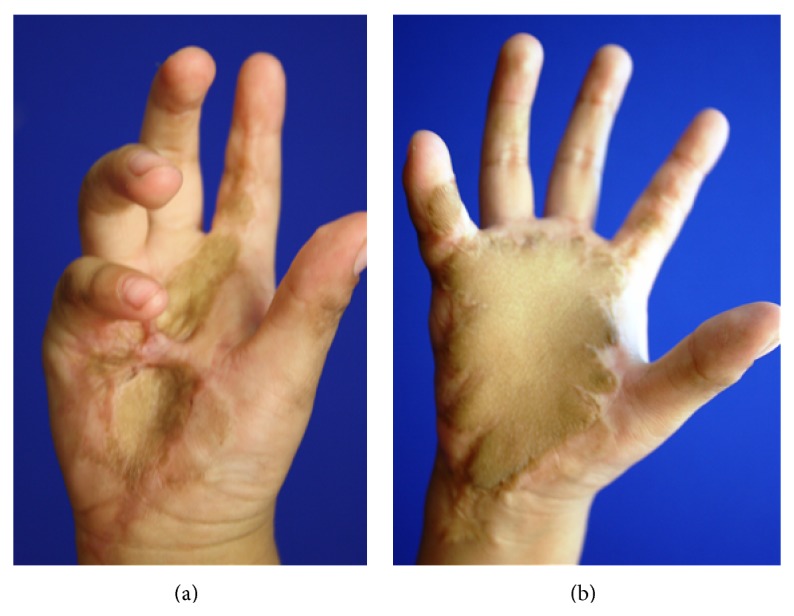
Before and after images of a 4-year-old child with burn sequelae on her dominant hand.

The aesthetic outcome was assessed using the Likert scale for evaluation of aesthetic results in free flaps, in which four main factors are evaluated on a numerical scale: general appearance, shape, color, and texture [9] (Figure 2).

The combined numerical score of the Likert scale (min, 4; max, 20) was classified as follows: 4 to 6, poor; 7 to 9, bad; 10 to 13, regular; 14 to 16, good; and 17 to 20, very good. This evaluation was performed for each patient by three plastic and reconstructive surgeons not involved in the research, an associated researcher (second-year resident), and a close relative. We averaged the scores and obtained a total that was used for classification within the ranges mentioned above.

In addition to evaluation of the overall aesthetic result, we analyzed each one of the four evaluated aesthetic aspects: general appearance, contour, color, and texture. These factors were compared with the features of a normal extremity on a scale of 1 to 5 in which 1 was the worst rating and 5 the best (1, strongly disagree; 2, disagree; 3, neither agree nor disagree; 4, agree; 5, strongly agree).

Functionality was evaluated by the Spanish validated version of the QuickDASH scale [10, 11], which assesses motor functions of the upper extremity using a simple questionnaire of 11 items. This value was then converted to a score of 0 to 100 using the formula described within the scale. This questionnaire was answered by the patient or, in the case of infants, a parent or guardian.

The data were validated using descriptive statistics (means, confidence intervals, and percentages) with SPSS version 19.0 statistical software. The study was approved by the Research Ethics Committee of our center, the Hospital General Dr. Manuel Gea Gonzalez. All procedures were in accordance with the Regulations of the General Health Law in the Field of Health Research.

## 3. Results

According to the patients analyzed, 6 were male (85.7%) and 1 was female (14.3%), with a mean age of 12.43 years (min: 2 and max: 28, confidence interval 95% (95% CI) 1.66–23.20), with a mean follow-up evaluation of 11.5 months (min: 7 and max: 18, 95% CI 7.62–15.53).

The mean flap thickness was 5 mm, and the mean size was 13 × 8.2 cm (min: 6 × 5 cm, max 26 × 15 cm), taking care to maintain a safe ratio around the perforating vessels to ensure survival of the flap (1 cm) (Table 2).  It is important to mention that the different areas of the defects to be reconstructed had the same dimensions of the designed flaps for each one.

The aesthetic outcome was assessed using the Likert scale for evaluation of aesthetic results in free flaps, and each patient was evaluated in the manner previously mentioned (Table 3).

The differences between the overall score of the evaluators means and their associated procedures were measured with the ANOVA (analysis of variance). Concordance among the first three evaluators was good (>0.61) and without statistically significant difference in the overall scores (ANOVA) of the rest of the evaluators (Table 4).

The global analysis results for each characteristic of the aesthetic evaluation scale are shown in Table 3.

Evaluation of general aesthetics revealed a mean of 15.4 (good), CI (95%): 1.50–2.20, with the value of each characteristic evaluated as follows: appearance: mean, 3.60 (CI (95%): 3.11–4.1); contour: mean, 3.85 (CI (95%): 3.28–4.42); color: mean, 3.91 (CI (95%): 3.47–4.35); and texture: mean, 3.97 (CI (95%): 3.62–4.31).

**Figure 3:**
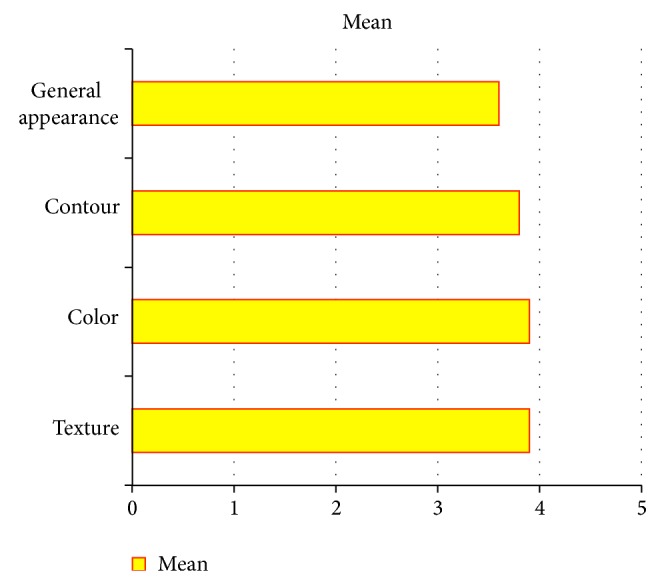
Aesthetic evaluation characteristics.

The best rated features were color and texture, with a mean score of 3.9; the worst rated feature was overall appearance, with a score of 3.6 (Figure 3, Table 5).

**Figure 4:**
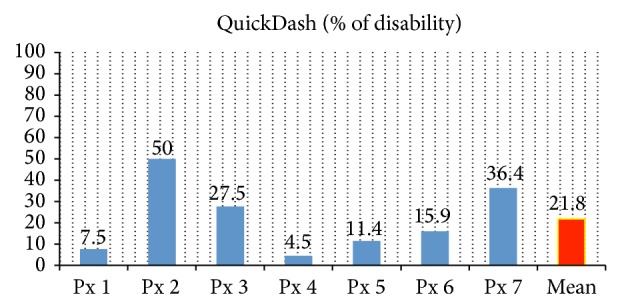
Rating of QuickDASH scale.

Functional aspects showed a mean of 21.88% (CI (95%): 6.37–37.39) as evaluated by the QuickDASH scale (Table 6, Figure 4). This percentage represents a low disability that means that most of the common uses of the upper limb, as grabbing or taking a pen or a glass of water, could be done.

Perception of pain and touch was present in 100% of patients. The temperatures (cold and heat) were positive in 85% of patients on the entire flap. Just in one patient (15%) the perception of both cold and heat was absent in the distal half of the flap.

Two-point discrimination showed a mean of 8.57 mm (CI (95%): 3.05–14.09) in the proximal half of the flap and a mean of 9.71 mm (CI (95%): 4.33–15.10) in the distal half. Note that the youngest patient (Px 3) failed to perform this evaluation because of the patient's difficulty in interpretation (Table 6).

## 4. Discussion

Thinning of the ALT free flap was described by Kimura et al. in 2011. They defined an ultrathin flap as a flap with a 6 mm thickness [12]. Our series is in line with this definition in which we achieved minimum and maximum thickness of 4 and 6 mm, respectively.

A clear understanding of the vascular anatomy of a perforator flap is mandatory, and the* perforasome* concept helps to achieve this understanding. Each perforator possesses a unique tridimensional vascular territory and can be linked by direct or indirect linking vessels to other perforators, increasing the potential size of a flap.

These linking vessels, as described by Taylor and Saint-Cyr, can be established by two mechanisms: (1) the direct linking vessels located in the suprafascial plexus and adipose layer and (2) the indirect linking vessels that depend on a low-pressure reflux phenomenon in the subdermal plexus. These systems also have communications between them to maintain the perfusion pressure. Their distribution is tridimensional and allows for differences in pressure, inversion of flow, and bidirectional flow to recruit adjacent perforasomes, first in the axial plane and later in the transverse plane [13, 14]. The significance of these concepts of perforator flow between perforators directly impacts flap design and survival. We believe that this helped us to achieve a 100% survival rate.

The innervation of the ALT flap has been widely studied. Innervation is heavily reliant on the femoral cutaneous nerve as the dominant nerve. There are two other well-identified but inconsistent branches: the superior and medial perforator nerves. They share territory with the lateral femorocutaneous nerve because of anastomoses between them [15].

An important factor in the neurorrhaphy is the axonal charge of the receptor nerve because it has a direct impact on the velocity of nerve regeneration. In a terminolateral neurorrhaphy, the axonal charge provided to the nerve is smaller, and the regeneration time is thus longer. This knowledge prompted us to perform terminoterminal neurorrhaphies, decreasing the reinnervation time.

The largest report of an ultrathin ALT flap was reported by Kimura et al. [12]. Their study involved 31 patients during a 6-year period. There were variations in width and length, but the average was 7.7 × 14.7 cm to preserve flap vascularity and survival. Our series had an average size of 13 × 8.2 cm, and every flap was designed to cover a specific defect. Thinning of the flap is a challenge for the surgeon, but it could be made easier by following general principles such as blunt dissection of the vascular pedicle, maintaining a minimum thickness of 3 to 4 mm, and designing the skin paddle with a minimum of 4.5 cm safe ratio around the vascular pedicle, for a total diameter of 9 cm. Following these principles will ensure the safety of the procedure, with the advantage of a one-stage reconstruction that allows a wide and early range of movement, especially in the hand and fingers.

Our series did not have a control group with which to compare our flap because of the presence of different types of defects, making it impossible to create an identical flap in every patient. However, based on the aesthetic scale for free flaps (Likert scale), we provided a good general contour and avoided numerous debulking procedures or liposuction to achieve a good appearance [15].

There is not enough evidence to conclude that flap thinning will compromise the vascularity of the flap. In addition, thinning will not affect the degree of reinnervation of the flap or the velocity of sensibility restoration in the reconstructed area, primarily when the dominant nerve of the flap is well identified. There are currently no reports on this relationship, and more specific studies are necessary to define the timing of nerve regeneration.

Postsurgical sensory evaluation is limited in pediatric patients because of difficulties related to the children's cooperation and interpretation of the two-point discrimination test, as in one of our cases. An excellent option in this circumstance is the neurophysiologic test known as somatosensorial evoked potentials [16]. However, the increased cost and requirement for human resources should be taken into account. These factors justified the absence of this test in our series. Otherwise, performance of this test should be considered when a sensory evaluation cannot be accurately performed for any reason (age, psychiatric illness, etc.). As previously mentioned and observed in our series, the optimal age for this reconstruction, with the exception of emergency cases, is >3 years; at this age, the patient is usually psychologically and physically able to cooperate with the evaluation.

Previous reports on the aesthetic evaluation of free flaps have been published. Parret et al. used the Likert scale to evaluate different types of free flaps for reconstruction of the dorsum of the hand. In their report, venous flaps provided the best cosmetic appearance among fascial, muscular, and fasciocutaneous flaps [9]. Our series included the entire hand, wrist, and forearm, unlike Parret's report, which focused only on dorsum defects. Another critical point is the size of our defects; unlike Parret, we did not use venous flaps. Previous reports mentioned the small size of the venous flap and that its vascularity depends on neovascularization from the arteriovenous shunt created within the flap; trying to increase its size could jeopardize the vascularity, producing venous congestion and necrosis [9, 17].

Making a comparison of our results with the publication of Parret, which makes a similar aesthetic assessment, we can say that our results are better in general appearance and contour than his fasciocutaneous flaps (3.6 versus 2.24 and 3.8 versus 1.95, resp.), better in color than his fascial, muscle, and fasciocutaneous flaps (3.9 versus 3.62, 3.76, and 2.52, resp.), and better in texture, also compared with his fascial, muscle, and fasciocutaneous flaps (3.9 versus 3.29, 3.48, and 3.1, resp.).

From the functional point of view, our assessment was made by the validated scale QuickDASH, which is a shortened version of the DASH scale to measure the degree of disability of the upper extremity (for its acronym in English, Disability of the Arm and Shoulder). This scale does not measure the degree of disability as mild, moderate, or severe. Instead, the result is expressed as a percentage of disability. According to the article published by Kovacs et al. [18], which is the largest series so far studied, with 118 patients, he performs a comparative analysis of the injured reconstructed limb with the healthy contralateral limb. According to these reports, we observed that the scale measured a diminishing result as the patient adapted to the conditions of his injured limb and was still able to perform more tasks as time passed, even without rehabilitation exercises. According to the average result in our study in the QuickDASH scale of 21.88%, with a mean follow-up of 11.57 months, we find a very low disability percentage in our patients, compared with those described in the international literature, and it is even better than those assessed by Kovacs, who concluded that the average rating of the DASH scale before 3 years after the injury is of 28.7% and is of 20.2% after 3 years.

An important difference in our report is the heterogeneous group of evaluators (three certified plastic surgeons, one second-year plastic surgery resident, and a first-degree relative of the patient), which provides a more objective result. In this study, we ruled out the patients' opinions because of the young age of many of the patients; otherwise, this may be studied in the future as a new line of investigation. The concordance among the three first evaluators was good (>0.61), and that between evaluators 4 and 5 was regular (0.54). Otherwise, after statistically analyzing the results of all of the evaluators (one-way ANOVA), no significant statistical differences were found (>0.5).

## 5. Conclusions

Simultaneous thinning and innervation of the ALT flap lead to a good cosmetic result and functional outcome with a low percentage of disability according to the rating scales in the present study. This technique may result in fewer surgeries, better recovery of motor and sensory function, and significant improvement in color and texture on the reconstructed tissue.

## Figures and Tables

**Table 1:** Demographic data, showing age, gender, anatomic region where the flap was inset, and the etiology.

Patient	Age	Gender	Upper limb defect	Etiology
1	3	Male	Dorsal side of the left hand	Burn sequelae
2	6	Male	Volar side of the distal right forearm and hand	Trauma sequelae
3	2	Male	Dorsal and lateral side of the distal left forearm	Acute trauma
4	27	Male	Dorsal side of the right hand	Burn sequelae
5	28	Male	Dorsal side of the distal right forearm	Acute trauma
6	18	Male	Volar side of the left 4th finger	Acute trauma
7	3	Female	Volar side of the right hand	Burn sequelae

**Table 2:** Anterolateral thigh flap characteristics among patients.

Px	Thickness flap (mm)	Length (cm)	Width (cm)
1	6	6	5
2	6	12	8
3	5	11	6
4	4	14	10
5	5	26	15
6	4	12	6
7	5	10	8

	Mean: 5 mm	Min: 6 cm Max: 26 cm Mean: 13	Min: 5 cm Max: 15 cm Mean: 8.2

Abbreviations: Px: patient, mm: millimeters, and cm: centimeters.

**Table 3:** Aesthetic evaluation.

Px	ESP 1	ESP 2	ESP 3	Relative	2nd Y.R.	Min/max	Mean	Qualification
1	19	20	20	20	15	15/20	**18.8**	**Very good**
2	17	14	19	13	11	11/19	**14.8**	**Good**
3	17	19	19	10	13	10/19	**15.6**	**Good**
4	14	16	16	14	13	13/16	**14.6**	**Good**
5	13	19	18	16	12	12/19	**15.6**	**Good**
6	14	16	18	13	11	11/18	**14.4**	**Good**
7	18	19	13	8	12	8/19	**14**	**Good**

Abbreviations: Px: patient; ESP: evaluating specialist; 2nd Y.R: second-year resident.

**Table 4:** ANOVA for evaluators.

Analysis by the evaluators (ANOVA)	
Evaluator 1 (specialist 1):	0.747
Evaluator 2 (specialist 2):	0.327
Evaluator 3 (specialist 3):	0.205
Evaluator 4 (2nd year resident):	0.681
Evaluator 5 (relative):	0.343
	(>*0.5*)

Abbreviations: Evaluator 1: specialist 1; evaluator 2: specialist 2; evaluator 3: specialist 3; evaluator 4: second-year resident; evaluator 5: relative.

**Table 5:** Aesthetic evaluation by characteristics.

Px	General appearance	Contour	Color	Texture	Mean patients
1	4.8	4.8	4.6	4.6	**4.7**
2	3.4	3.4	4	4	**3.7**
3	4	3.4	4.4	3.8	**3.9**
4	3.4	3.8	3.4	4	**3.6**
5	3.6	4.6	3.6	3.8	**3.9**
6	3	3.2	4	4.2	**3.6**
7	3.4	3.8	3.4	3.4	**3.5**
Mean characters	**3.6**	**3.8**	**3.9**	**3.9**	***3.8***

Abbreviations: Px: patient.

**Table 6:** Functional evaluation.

Px	QuickDASH (% of disability)	Touch	Pain	temp hot	temp cold	2-point disc. proximal (mm)	2-point disc. distal (mm)
1	7.5	+	+	+	+	8	10
2	50	+	+	+	+	10	10
3	27.5	+	+	+	+	Unable	
4	4.5	+	+	+	+	6	10
5	11.4	+	+	+	+	8	10
6	15.9	+	+	+ (1/2 prox)	+ (1/2 prox)	20	20
7	36.4	+	+	+	+	8	8

	Mean: 21.8					Mean: 8.57 mm	Mean: 9.71 mm

Abbreviations: Px: patient; temp: temperature; prox: proximal.

## References

[1] Song Y.-G., Chen G.-Z., Song Y.-L. (1984). The free thigh flap: a new free flap concept based on the septocutaneous artery. *British Journal of Plastic Surgery*.

[2] Kimata Y., Uchiyama K., Ebihara S., Nakatsuka T., Harii K. (1998). Anatomic variations and technical problems of the anterolateral thigh flap: a report of 74 cases. *Plastic and Reconstructive Surgery*.

[3] Demirkan F., Chen H.-C., Wei F.-C., Chen H.-H., Jung S.-G., Hau S.-P., Liao C.-T. (2000). The versatile anterolateral thigh flap: a musculocutaneous flap in disguise in head and neck reconstruction. *British Journal of Plastic Surgery*.

[4] Ribuffo D., Cigna E., Gargano F., Spalvieri C., Scuderi N. (2005). The innervated anterolateral thigh flap: anatomical study and clinical implications. *Plastic and Reconstructive Surgery*.

[5] Nojima K., Brown S. A., Acikel C., Arbique G., Ozturk S., Chao J., Kurihara K., Rohrich R. J. (2005). Defining vascular supply and territory of thinned perforator flaps: part I. Anterolateral thigh perforator flap. *Plastic and Reconstructive Surgery*.

[6] Adani R., Tarallo L., Marcoccio I., Cipriani R., Gelati C., Innocenti M. (2005). Hand reconstruction using the thin anterolateral thigh flap. *Plastic & Reconstructive Surgery*.

[7] Jeon B.-J., Lim S.-Y., Pyon J.-K., Bang S.-I., Oh K. S., Mun G.-H. (2011). Secondary extremity reconstruction with free perforator flaps for aesthetic purposes. *Journal of Plastic, Reconstructive & Aesthetic Surgery*.

[8] Alkureishi L. W. T., Shaw-Dunn J., Ross G. L. (2003). Effects of thinning the anterolateral thigh flap on the blood supply to the skin. *British Journal of Plastic Surgery*.

[9] Parrett B. M., Bou-Merhi J. S., Buntic R. F., Safa B., Buncke G. M., Brooks D. (2010). Refining outcomes in dorsal hand coverage: consideration of aesthetics and fonor-Site morbidity. *Plastic and Reconstructive Surgery*.

[10] Institute for Work & Health QuickDASH scale. Versión Española (España).

[11] http://www.dash.iwh.on.ca/assets/images/pdfs/quickdash_info_2010.pdf.

[12] Kimura N., Satoh K., Hasumi T., Ostuka T. (2001). Clinical application of the free thin anterolateral thigh flap in 31 consecutive patients. *Plastic and Reconstructive Surgery*.

[13] Taylor G. I. (2003). The angiosomes of the body and their supply to perforator flaps. *Clinics in Plastic Surgery*.

[14] Saint-Cyr M., Wong C., Schaverien M., Mojallal A., Rohrich R. J. (2009). The perforasome theory: vascular anatomy and clinical implications. *Plastic and Reconstructive Surgery*.

[15] Askouni E. P., Topping A., Ball S., Hettiaratchy S., Nanchahal J., Jain A. (2012). Outcomes of anterolateral thigh free flap thinning using liposuction following lower limb trauma. *Journal of Plastic, Reconstructive and Aesthetic Surgery*.

[16] Papazian O., Alfonso I., García V. F. (2002). Evaluación neurofisiológica de los niños con neuropatías periféricas. *Revista de Neurologia*.

[17] Lin Y.-T., Henry S. L., Lin C.-H., Lee H.-Y., Lin W.-N., Wei F.-C. (2010). The shunt-restricted arterialized venous flap for hand/digit reconstruction: enhanced perfusion, decreased congestion, and improved reliability. *Journal of Trauma: Injury Infection & Critical Care*.

[18] Kovacs L., Grob M., Zimmermann A., Eder M., Herschbach P., Henrich G., Zimmer R., Biemer E., Papadopulos N. A. (2011). Quality of life after severe hand injury. *Journal of Plastic, Reconstructive and Aesthetic Surgery*.

